# Psychopathic tendency in violent offenders is associated with reduced aversive Pavlovian inhibition of behavior and associated striatal BOLD signal

**DOI:** 10.3389/fnbeh.2022.963776

**Published:** 2022-10-14

**Authors:** Dirk E. M. Geurts, Katinka von Borries, Quentin J. M. Huys, Berend H. Bulten, Robbert-Jan Verkes, Roshan Cools

**Affiliations:** ^1^Centre for Cognitive Neuroimaging, Donders Institute for Brain, Cognition and Behaviour, Radboud University, Nijmegen, Netherlands; ^2^Department of Psychiatry, Radboud University Medical Center, Nijmegen, Netherlands; ^3^Pompestichting Center for Forensic Psychiatry, Pro Persona Mental Health, Nijmegen, Netherlands; ^4^Division of Psychiatry and Max Planck UCL Centre for Computational Psychiatry and Ageing Research, Mental Health Neuroscience Department, Institute of Neurology, University College London, London, United Kingdom

**Keywords:** psychopathy, Pavlovian-to-instrumental transfer, inhibition, fMRI, amygdala, caudate, putamen

## Abstract

**Background:**

Violent offenders with psychopathic tendencies are characterized by instrumental, i.e., planned, callous, and unemotional (aggressive) behavior and have been shown to exhibit abnormal aversive processing. However, the consequences of abnormal aversive processing for instrumental action and associated neural mechanisms are unclear.

**Materials and methods:**

Here we address this issue by using event-related functional magnetic resonance imaging (fMRI) in 15 violent offenders with high psychopathic tendencies and 18 matched controls during the performance of an aversive Pavlovian-to-instrumental transfer paradigm. This paradigm allowed us to assess the degree to which aversive Pavlovian cues affect instrumental action and associated neural signaling.

**Results:**

Psychopathic tendency scores were associated with an attenuation of aversive Pavlovian inhibition of instrumental action. Moreover, exploratory analyses revealed an anomalous positive association between aversive inhibition of action and aversive inhibition of BOLD signal in the caudate nucleus of violent offenders with psychopathic tendencies. In addition, psychopathic tendency also correlated positively with amygdala reactivity during aversive versus neutral cues in Pavlovian training.

**Conclusion:**

These findings strengthen the hypothesis that psychopathic tendencies in violent offenders are related to abnormal impact of aversive processing on instrumental behavior. The neural effects raise the possibility that this reflects deficient transfer of aversive Pavlovian inhibitory biases onto neural systems that implement instrumental action, including the caudate nucleus.

## Introduction

Instrumental decision making is susceptible to emotional/affective influences (Estes and Skinner, 1941; Damasio, 1997). Evidence suggests that this affective biasing of action selection can reflect an interaction between distinct behavioral control systems (Cardinal et al., 2002; Dayan et al., 2006; Kahneman and Frederick, 2007). For example, instrumentally controlled action selection is well established to be sensitive to biasing by a Pavlovian or hardwired “affective” system that regulates innately specified responses to aversive stimuli (Dayan and Seymour, 2013; Guitart-Masip et al., 2014). This Pavlovian system allows agents to control behavior through strategies that have been learnt across a lifetime and/or generations to be adaptive and thus to be generalizable to novel situations. Examples of such strategies are our tendencies to promote approach (and suppress withdarawal) actions when facing reward or to suppress approach (and potentiate withdrawal) actions when facing punishment. These strategies allow us to circumvent more expensive, rational instrumental (context-appropriate) calculations and to make judgments quickly and efficiently. However, they can also contribute to maladaptive behavior.

Anomalies in the interaction between these Pavlovian and instrumental control systems have been proposed to account for behavioral impairments seen in a wide variety of neuropsychiatric disorders (Dayan et al., 2006; Heinz et al., 2016; Huys et al., 2016; Hallquist et al., 2018; Nord et al., 2018; Chen et al., 2021). Here we focus on the high end of a psychiatric dimension (Patrick, 2022) that imposes a large burden on individual victims and society as a whole: psychopathic tendency. Specifically we study the interaction between Pavlovian and instrumental control systems in a group of violent offenders with high degrees of psychopathic tendency and a group of matched healthy controls. Psychopathic tendency is characterized by affective and behavioral anomalies (De Brito et al., 2021) and has been associated, in violent offenders, with “instrumental aggression” (i.e., callous and unemotional aggressive behavior) and high rates of recidivism even after prison sentences (Hare, 2003; Leistico et al., 2008; Warren and Burnette, 2013). Given our interest in psychopathic tendency, we employed the Psychopathy Checklist—revised (PCL-R) (Hare, 2003) as a psychological assessment tool to quantify, in each violent offenders, the degree of psychopathic tendency. The degree to which variation in Pavlovian-instrumental interaction varies with regard to individual differences in the PCL-R score were then assessed using correlational analyses.

A core feature of the crimes committed by violent offenders with psychopathic tendency is their “instrumentality,” i.e., their planned, callous, and unemotional nature (Blair, 2001; De Brito et al., 2021). These crimes are premeditated and committed to achieve a desired goal at the expense of others. Despite the centrality of such callous and unemotional action in clinical observations and in elaborate cognitive models of psychopathic tendency [e.g., the violence inhibition model (Blair, 2005)], neuroscientific research on the mechanisms of instrumental action (i.e., actions planned to obtain a certain outcome) in the face of aversive cues is scarce. So far, the neuroscience of psychopathic tendency has focused mainly on reduced affective (primarily aversive) processing *per se* and associated neural signals, for example, in limbic circuitry (Brook et al., 2013). There is evidence (albeit in small samples) that people with psychopathic tendency respond normally to unconditioned aversive Pavlovian stimuli (US), but that their psychophysiological responses to conditioned aversive stimuli (CS) are compromised (Flor et al., 2002; Veit et al., 2002; Birbaumer et al., 2005; Rothemund et al., 2012; Schultz et al., 2016). However, it is unclear how such a deficiency in aversive information processing is related to the behavioral abnormalities of psychopathic tendency. Studies focusing on affective anomalies *per se* do not provide insight in the behavioral deficits that might stem from these affective anomalies. We set up the current study to test directly the hypothesis that psychopathic tendency is associated not only with abnormal aversive processing *per se*, but rather also with reduced transfer of aversive Pavlovian biases to instrumental behavior. We thus addressed one instance of the more general proposal that neuropsychiatric abnormality is associated with an absence of Pavlovian solutions to behavioral control.

The present study was conducted around the same time as another study we performed with violent offenders (Ly et al., 2016) to test this same hypothesis. This prior study indeed demonstrated reduced potentiation of instrumental avoidance (versus approach) actions by aversive angry (versus appetitive happy) faces in a group of violent offenders compared with a group of matched controls. The added value of the present study is threefold. First, we provide a conceptual replication, thus increasing the construct validity of this prior finding by showing reduced impact of aversive Pavlovian cues on instrumental action in a different group of violent offenders with high levels of psychopathic tendencies, using a different paradigm. Notably, by including a neutral Pavlovian cue, this paradigm allowed us to establish that the altered impact of Pavlovian cues was due to reductions in aversive bias instead of increases in appetitive bias. Second, the present study addresses neural BOLD responses associated with aversive Pavlovian conditioning and the influence of aversive Pavlovian cues on instrumental behavior in violent offenders, showing a key role for the striatum in abnormal Pavlovian control of behavior. Finally, we demonstrate that the behavioral and neural changes are a function of individual differences in psychopathic tendency.

In our previous study, affective biases by facial cues were indexed during one and the same instrumental learning phase (Ly et al., 2016). By contrast, the paradigm employed here comprised three separate phases, allowing us to disentangle (i) instrumental action learning impairment, indexed during a first phase, from (ii) changes in the learning of, and responsiveness to Pavlovian cues themselves, indexed during a second Pavlovian conditioning phase, and (ii) changes in the key process of interest: Pavlovian-to-instrumental transfer, indexed during a final task phase. This key PIT process of interest was anticipated, based on prior work (Huys et al., 2011, 2016; Geurts et al., 2013a), to surface, across all participants, as potentiation of aversive instrumental withdrawal actions, but suppression of instrumental approach actions in the context of aversive Pavlovian cues, i.e., stimuli that predict aversive outcomes. Following our prior observation (Ly et al., 2016), violent offenders were expected to exhibit reduced impact of aversive cues on both types of instrumental action and we assess specifically whether this surfaces in a psychopathic tendency-dependent manner. Thus, we predicted that they exhibit reduced aversive inhibition of approach as well as reduced aversive potentiation of withdrawal actions.

Next, we assessed the neural mechanisms underlying the aversive PIT effects. Animal and human studies consistently implicate frontostriatal brain regions in instrumental action, especially the dorsomedial (caudate nucleus) and dorsolateral (putamen) parts of the striatum and the ventromedial regions of the prefrontal cortex (Tricomi et al., 2004; Valentin et al., 2007; Balleine and O’Doherty, 2009; Wunderlich et al., 2009; Dolan and Dayan, 2013). In addition, affective information is known to influence instrumental actions *via* the amygdala (Cardinal et al., 2002; Talmi et al., 2008; Balleine and O’Doherty, 2009; Prevost et al., 2012; Geurts et al., 2013a; Ly et al., 2014), and extensive evidence implicates dysfunction of the amygdala in people with psychopathic tendency (Veit et al., 2002; Birbaumer et al., 2005; Blair, 2008; Glenn and Raine, 2009; Moul et al., 2012). Thus, we anticipated, that violent offenders with psychopathic tendency would exhibit changes in aversive cue-related BOLD signal in the amygdala as well as differential aversive modulation of instrumental action-related signals in frontal and striatal brain regions. To this end, we focused our primary analyses on the striatum, ventromedial prefrontal cortex, and the amygdala.

## Materials and methods

### Subjects

Eighteen male violent offenders with psychopathic tendency (three left-handed) volunteered and were selected based on available information about clinical status and history from an in-patient population of a forensic hospital (Supplementary material and methods). All received a court-imposed placement under a hospital order with imprisonment for committing violence offenses repeatedly, including murder, slaughter, battery, rape, while suffering from psychiatric illness or disorder. The violent offenders all had a score of ≥26 on the Hare Psychopathy Check List-Revised (PCL-R) (Hare, 2003; Table 1). Additionally, twenty healthy men matched for age and IQ without criminal records or a history of psychiatric disorders were recruited from among the employees of the same hospital by advertisement. Participants in both groups were screened for drug use and for medical/neurological history (Supplementary material and methods and Table 1). Considering the particularities of the population, the testing environment, and the time period when testing was possible, these were the maximum numbers of inclusion.

**TABLE 1:** Group characteristics (mean, standard deviation) of the group of violent offenders with psychopathic tendency and healthy matched control subjects.

	Violent offenders with psychopathic tendency (*n* = 15)	Healthy controls (*n* = 18)	Statistics (*P*-value)
Age	40.2 (9.1)	41.2 (10.4)	0.78
IQ (NLV)	101.7 (8.8)	101.5 (8.7)	0.96
PCL-R total	30.7 (4.0)	–	–
PCL–R factor 1	11.9 (2.9)	–	–
PCL-R factor 2	13.9 (2.1)	–	–

Exclusion criteria for both groups were: (i) Use of alcohol more than 3 units/day during the week preceding the experimental measure and use of alcohol within 24 h of the measurement.

(ii) Use of cannabis or other illicit drugs within the week before measurement and use of psychotropic medication other than oxazepam during the 5 days before measurement.

(iii) Use of oxazepam within 12 h before measurement.

(iv) Smoking within 3 h before measurement.

(v) History of trauma capitis, visual and auditory disorders, neurological disorders, first degree relative with any relevant neurological disorders.

Following previous studies (Brazil et al., 2009; von Borries et al., 2009), exclusion criteria were all major Axis-I and Axis-II disorders except for cluster B personality disorders in violent offenders, psychotropic medication, cannabis or other drug use 1 week before, alcohol or oxazepam use within 24 h before experiment, visual disorder, and neurological disorder. Furthermore, individuals not eligible for MRI scanning were excluded.

All participants received oral and written information about the experiment and gave written informed consent. They received payment as a reimbursement for participation. The study was performed in accordance with the Declaration of Helsinki and approved by the local ethical committee (NL30545.091.09).

Two violent offenders withdrew from participation and one violent offender was excluded because of excessive head movement (more than twice the voxel size). Two non-criminal healthy controls were excluded because their behavioral data suggested they did not follow the instructions during the PIT stage [despite instructions to play the instrumental game (see paradigm) these participants determined their actions solely on the Pavlovian CS, but never on the instrumental stimuli in more than half of the trials: 58 and 83% resp., compared with on average 1%, range 0–17%, for all other participants]. Moreover, due to technical issues with the scanner and excessive head movement only one of two runs could be analyzed for one healthy control and two violent offenders. Thus, we analyzed datasets of 15 violent offenders and 18 healthy controls.

### Pavlovian-instrumental transfer paradigm

**FIGURE 1:**
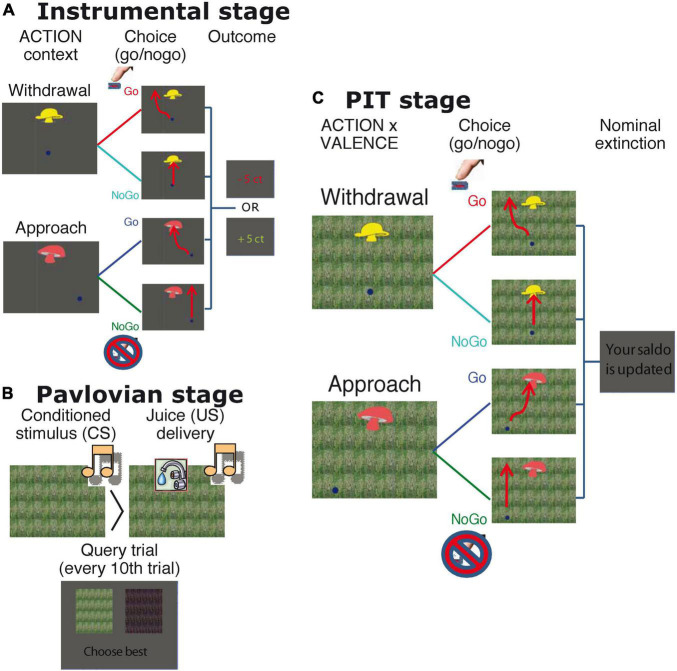
Task details. **(A)** Instrumental stage. Trials started with the appearance of the instrumental stimulus at the top center of the screen and of a dot at the bottom of the screen. In approach trials, the dot started either on the left or on the right bottom side of the screen. Participants could choose to do nothing (approach-no-go), in which case the dot would wiggle past the instrumental stimulus. Alternatively, they could push the button repeatedly to steer the dot through the instrumental stimulus (approach-go). In withdrawal trials, the dot started centrally at the bottom beneath the instrumental stimulus. Participants could choose to push the button repeatedly to avoid moving through instrumental stimulus (withdrawal-go) or to do nothing (withdrawal-no-go). The four possible trajectories are drawn in the figure (red lines). If the dot entered the goal region, then the instrumental stimulus was collected. After the dot moved outside the window feedback was provided. Thus, there were 2 ACTION contexts (approach and withdrawal), with each 4 different instrumental stimuli, with 2 stimuli resulting more often in reward after a go-action and 2 resulting more often in reward after a no-go. Each stimulus was presented 10 times, resulting in (2 × 4 × 10=) 80 instrumental trials (divided in mini blocks of 8 withdrawal or approach trials). The straight line just to one side of the instrumental stimulus was a reflecting boundary that the dot could not cross. Timings were as follows: Instrumental stimuli were presented for 2.5 s, during which responses were collected. After 2.5 s, feedback was presented for 1 s. The intertrial interval (ITI) was 1 s (blank screen). **(B)** Pavlovian stage. Each Pavlovian conditioned stimuli (CS) was presented 20 times, and for each session there was a separate set of three stimuli. Stimulus presentation order was fully randomized across participants. Stimulus duration was 4.5 s, and juice delivery (2 ml) occurred between 0 and 1.5 s after stimulus onset. The ITI was 1 s. Query trials were presented after every 10 Pavlovian trials. On these trials, participants chose one of the two presented Pavlovian (audiovisual) stimuli (presented for 2 s; ITI 0.5 s) without any feedback. **(C)** PIT stage. The PIT stage paralleled the instrumental training, except that Pavlovian CSs tiled the background. Each instrumental stimulus was presented 12 times and each Pavlovian CS was presented 32 times, counterbalanced across the different instrumental stimuli. No outcomes were presented, but participants were instructed that their choices counted toward the final total. Participants were explicitly instructed that the juices were collected outside the scanner, and they agreed before the start of the experiment to drink them afterward. Timing of one trial was as follows: 250 ms after the onset of the Pavlovian stimulus, the instrumental stimulus (and dot) was overlaid on top of this Pavlovian stimulus. Duration of the instrumental stimulus was 2.5 s; duration of the Pavlovian stimulus was 2.75 s. Upon offset of both stimuli, feedback was presented, which consisted only of the words “Balance is updated” (duration = 1 s, ITI = 1 s). Note that there were two runs in which all three stages (with new independent Pavlovian and instrumental stimuli) were assessed.

Subjects performed a computerized task to assess aversive PIT (Geurts et al., 2013a; Supplementary material; Figure 1). The experiment consisted of three stages: (1) instrumental, (2) Pavlovian, and (3) PIT stage. The instrumental stage contained two Action Contexts: (i) a context in which the active response led to an approach action and (ii) another in which the active response led to a withdrawal action (Figure 1A). In the approach Action Context subjects learned through monetary feedback (wins and losses) whether to “collect” the instrumental stimulus (approach-go) or not (approach-no-go). In the withdrawal Action Context they learned to avoid collecting instrumental stimuli (withdrawal-go) or not (withdrawal-no-go). Instrumental stimuli were randomly assigned to one of the four trial types. Thus, in both the approach and withdrawal Action Contexts, there were two go-stimuli, which yielded reward more often (i.e., ∼85% of the cases) after active responses (and punishment after not responding), and two nogo-stimuli, which yielded reward more often (i.e., also ∼85% of the cases) after not responding (and punishment after go-responding).

The second, Pavlovian stage consisted of repeated presentation of three audiovisual stimuli (Figure 1B): The appetitive and aversive conditioned stimuli (CS) were followed, respectively, by appetitive or aversive juice (i.e., the unconditioned stimuli Uss) on 50% of trials. The neutral CS resulted in no outcome. The appetitive juice was based on subjective preference for apple, orange, or strawberry lemonade. The aversive juice was a bitter magnesium sulfate solution (0.3M). Conditioning was assessed in two ways: (1) subjects indicated the degree to which they liked each of the CSs (and USs) by use of visual analog scales (VAS), before and after the experiment; (2) subjects chose one of the two presented Pavlovian stimuli (presented for 2 s; ITI 0.5 s) in extinction on 12 interspersed query trials.

In the third (PIT) stage stimulus presentation was the same as in the instrumental stage, except that Pavlovian stimuli tiled the background from 250 ms before (Larson et al., 2013) and no outcomes were presented (Figure 1C). Subjects were instructed that their choices counted toward the final monetary total, and that the juices associated with the Pavlovian outcomes were collected outside the scanner for them to drink afterward. There were two independent runs which each comprised different stimuli/CSs. Both runs included all three stages, and were separated by a 2-min break.

### Image acquisition

Whole-brain imaging was performed on a 3 Tesla MR scanner (Magnetrom Trio Tim, Siemens Medical Systems, Erlangen, Germany). Functional data were obtained using a multi-echo gradient T2*-weighted echo-planar scanning sequence (Poser et al., 2006; Supplementary material).

### Analyses

#### Behavioral data analysis

In keeping with our research aims we assessed group differences as well as parametric associations with PCL-R score. These scores were only available for the violent offenders. The behavioral data were analyzed using the statistic software SPSS 16.0 and Matlab^®^ 2009b.

##### Instrumental training

First, the proportion of correct responses was calculated for the first ten and last ten trials for each of the four trial types (covering all 80 instrumental trials). To assess whether subjects learned to make the correct choice, data were averaged across sessions and submitted to a repeated measures analysis of variance (rmANOVA) with Time (beginning/end of instrumental training), Action Context (approach/withdrawal) and Response (go/nogo) as within-subject and Group (healthy controls/violent offenders) as between-subject factor. Second, we assessed whether the learned behavior generalized to the PIT stage. Therefore, the factor Time was changed to include three levels: the end of the instrumental training and the beginning and the end of the PIT stage.

##### Pavlovian conditioning

Non-parametric tests were used to assess the proportion of correct responses on Pavlovian query trials and pre- and post-conditioning VAS ratings of the CS, because data were not distributed normally.

##### Pavlovian-instrumental transfer

The behavioral outcome measure was proportion of go actions, *p*(go), as a function of trial type (i.e., Action Context and CS Valence). Effects of CS Valence and Action on *p*(go) reflect PIT effects on choice. This dependent variable was first averaged across runs before it were submitted to an rmANOVA with Action Context (approach/withdrawal), and CS Valence(neutral/aversive) as within-subject factors and Group (healthy controls versus violent offenders) as a between-subject factor. Note that we focused our analyses on aversive PIT, based on our hypothesis (see Section “Introduction”) and on our previous work (*n* = 33) showing that the current paradigm was not sensitive to (and therefore not valid to assess) appetitive PIT [neutral vs. appetitive (Geurts et al., 2013a), Supplementary results]. The PCL-R-score was added as a covariate to assess its association with aversive PIT.

#### Functional magnetic resonance imaging analysis

Functional magnetic resonance imaging analysis was performed with SPM5 software (Wellcome Trust Centre for Cognitive Neuroimaging, London, UK). Pre-processing steps and first-level fMRI analysis were exactly as described by Geurts et al. (2013a): Pre-processing steps included applying a PAID-weight algorithm (Poser et al., 2006) to combine the different echoes, slice-time correction, coregistration, normalization based on parameters estimated through segmentation of the structural images, and smoothing.

The primary analysis was restricted to the PIT-stage. At the subject level a general linear model (GLM) was specified with 6 main regressors (4 of interest) representing the onset of the six different PIT trials of this paradigm [Action Context (approach/withdrawal) − Valence(appetitive/neutral/aversive)]. For each main regressor two additional parametric regressors were added (Büchel et al., 1996): The PIT-regressor (Talmi et al., 2008) was a parametric modulator of BOLD responses by the number of button presses per trial. A further parametric regressor contained the expectation associated with each instrumental stimulus (the *Q*-value) per trial as estimated from a model-based analysis of behavior (Huys et al., 2011). This was done based on prior data showing that BOLD signal in the prefrontal cortex and striatum, our regions of interest, covary with instrumental action value (e.g., Valentin et al., 2007; Wunderlich et al., 2009). As such, this approach maximized the degree to which our GLM captured variability in relevant BOLD signal. Furthermore, realignment parameters were added, high-pass filtering (128 s) was applied and parameter estimates were obtained by maximum-likelihood estimation (AR1).

The parameter estimates for the 4 parametric PIT-regressors were used in a 2 × 2 × 2 rmANOVA at the group-level (with random effects) with Action Context(approach/withdrawal) and Valence (neutral/aversive) as within-subject factors and GROUP (healthy controls/Vos) as between-subjects factor. Planned contrasts were the same as in Geurts et al. (2013a), but now assessed as a function of Group: [aversive-neutral] to reveal regions involved in aversive PIT across Action Contexts, and [(approach aversive-approach neutral)–(withdrawal aversive- withdrawal neutral)] to reveal regions involved in action-specific aversive PIT, and [approach–withdrawal] to reveal action-specific regions.

To capture group differences in brain-behavior associations, beyond those related to trial-by-trial variation, we contrasted the main regressors (Talmi et al., 2008; Geurts et al., 2013a) at the subject-level to calculate the main effect of Valence [(approach aversive + withdrawal aversive)–(approach neutral + withdrawal neutral)] and an interaction between Valence and Action Context [(approach aversive–approach neutral)–(withdrawal aversive–withdrawal neutral)]. The resulting individual contrasts were then used in a two-sample *t*-test at the group-level with behavioural aversive PIT-effects [*p*(go)] as a covariate for each group separately enabling comparison between groups. Thus, these analyses reveal regions, on a subject-by-subject basis, in which CS Valence-dependent BOLD signal change during the PIT stage was associated with aversive PIT. This association was assessed as a function of Action Context and Group. These analyses were repeated with PCL-R score (instead of behavioral PIT) as a covariate to assess whether CS Valence dependent BOLD signal change during the PIT stage was associated with psychopathy severity.

Next, additional analyses were performed to assess whether positive behavioral and fMRI findings from the PIT stage could be explained by BOLD signal change in the Pavlovian conditioning stage. Thus, we analyzed CS Valence-dependent BOLD signal change during the Pavlovian training phase as a function of individual differences in PCL-R score, aversive PIT and neural signaling in the caudate nucleus (see Section “Results”) during the PIT stage (each inserted as a covariate in three separate whole-brain analyses of CS Valence-related signals during the Pavlovian training phase).

First, at the subject level, a GLM was specified with six main regressors of interest representing the onset of the CS trials (during which no US was presented) in the beginning and the end of the conditioning stage: Valence (appetitive/neutral/aversive) × Time (early/late). This latter distinction between early and late acquisition, was based on evidence of rapid habituation of the responses in the amygdala during conditioning (Birbaumer et al., 2005). Early trials were the first three trials following the first US presentation for aversive and appetitive CS trials. For the neutral CS, the early trials were the first three presentations and the late trials were all the remaining CS presentations thereafter. To capture the other parts of the Pavlovian training, four regressors were added: for appetitive and aversive US onsets, for juice delivery onset (duration 2 s) and for the query trial onset (duration 2 s). Realignment and high-pass filtering was applied as before. Parameter estimates were obtained by maximum-likelihood estimation (AR1). We calculated the main effect of CS Valence (i.e., aversive-neutral) at the subject level for early, late and overall [early + late] conditioning and correlated the effects at the group-level (one sample *t*-test with covariate) with the PCL-R score; behavioral aversive PIT; and the extracted betas of the caudate nucleus as covariates of interest.

##### Regions of interest analysis

We report those effects that survive family wise error (FWE) correction for multiple comparisons across the whole brain (*P*_WB_ < 0.05, voxel-level) or regions of interest (ROIs, see Section “Introduction” for rationale): Following exactly the same procedure as in our previous work (Geurts et al., 2013a) the bilateral amygdala, caudate nucleus and putamen were defined using the automated anatomical labeling atlas in the WFU PickAtlas toolbox (Tzourio-Mazoyer et al., 2002). The bilateral nucleus accumbens was segmented for each participant using the FSL FIRST segmentation tool (Patenaude et al., 2011). These individual segments were then overlaid onto each other, generating one nucleus accumbens for the group (cf. Geurts et al., 2013a). Again following this prior work, we used the action-specific (approach > withdrawal) activation cluster [*p* < 0.001 uncorrected; peak voxel MNI-coordinates: (−8, 36, −8)] revealed by this previous PIT study to assess action specific signal in the vmPFC. The left and right elements of the bilateral volumes of interest were combined using Marsbar™ (Brett et al., 2002). Masks of the ROIs can be found in the Supplementary material.

## Results

### Behavioral data

#### Instrumental and Pavlovian stage

Healthy controls tended to learn faster than the violent offenders during instrumental training {Group × Time *F*_(1_,_31)_ = 4.3, *p* = 0.072; note, however, that they did not differ from violent offenders in terms of instrumental performance during the PIT stage [main effect of Group: *F*_(1_,_31)_ = 2.0, *P* = 0.17]}. There were no other relevant group differences in the instrumental and Pavlovian stages of the PIT paradigm (Supplementary material).

#### Pavlovian-instrumental transfer

**FIGURE 2:**
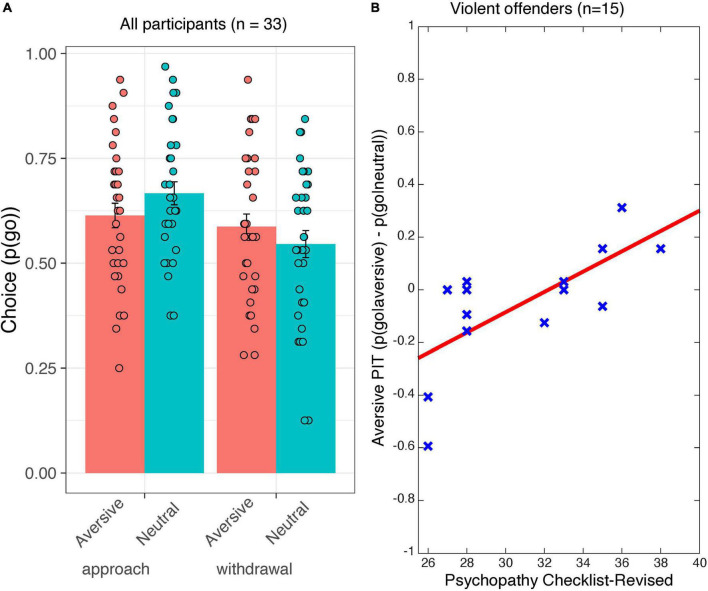
Behavioral data from the Pavlovian-instrumental transfer stage. Shown are **(A)** choice [*p*(go)] as a function of Action Context (approach and withdrawal) and Valence (aversive/neutral) for all participants. Error bars represent standard errors of the mean. **(B)** The correlation between aversive PIT [i.e., *p*(go| aversive)–*p*(go| neutral)] and psychopathy severity (in terms of the psychopathy checklist–revised total score). The red line is the ordinary least square trend line. Each cross represents an individual data point.

Data revealed the expected action-specific PIT effects across groups (Huys et al., 2011; cf. Geurts et al., 2013a): Aversive stimuli inhibited approach, but promoted withdrawal [Figure 2A; interaction Action Context(approach/withdrawal) × Valence(neutral/aversive): *F*_(1_,_31)_ = 8.6, *p* = 0.006; Valence during approach: *F*_(1_,_31)_ = 4.7, *p* = 0.037; Valence during withdrawal: *F*_(1_,_31)_ = 4.3, *p* = 0.046]. Subjects made more go-responses in the approach than in the withdrawal context overall [main Action Context effect: *F*_(1_,_31)_ = 9.9, *p* = 0.004]. However, in contrast to our expectation no significant main effect of or interaction with Group was found (*F* < 2.4, *p* > 0.161, Supplementary Table 2).

Regarding individual differences in psychopathic tendencies, we found that higher PCL-R scores were associated with less aversive inhibition, in a manner that was action-non-specific [interaction PCL-R score × Valence(neutral/aversive): *F*_(1_,_13)_ = 12.6, *p* = 0.004; Figure 2B]. Thus, higher PCL-R scores were associated with reduced aversive inhibition of approach actions [simple effects for approach block only: (*p*(go| aversive&approach)–*p*(go| neutral&approach)) × PCL-R score: *F*_(1_,_13)_ = 5.6, *p* = 0.035)] and a tendency toward increased aversively motivated withdrawal actions [simple effects for withdrawal block only: (*p*(go| aversive & withdrawal) – *p*(go| neutral & withdrawal)) × PCL-R score: *F*_(1_,_13)_ = 4.3, *p* = 0.059].

### Imaging data

In contrast to our hypothesis, the neural responses during aversive PIT did not differ between groups [no significant Action Context (approach/withdrawal) × Valence (neutral/aversive) × Group (violent offenders/healthy controls) interactions]. Action-specific signals (approach vs. withdrawal) across CS Valence were found in the precuneus [(12, −78, 6), *k* = 5453, *Z* = 7.02, *p*_FWE_ < 0.001, whole brain corrected], lingual [(10, −52, 52), *k* = 310, *Z* = 5.50, *p*_FWE_ = 0.001, whole brain corrected] and middle occipital gyrus [(34, −88, 2), *k* = 159, *Z* = 4.98, *p*_FWE_ = 0.016, whole brain corrected].

Moreover, within the group of violent offenders there was no significant effect of psychopathic tendency on BOLD signal during aversive PIT at the whole brain or in the pre-specified regions of interest.

**FIGURE 3:**
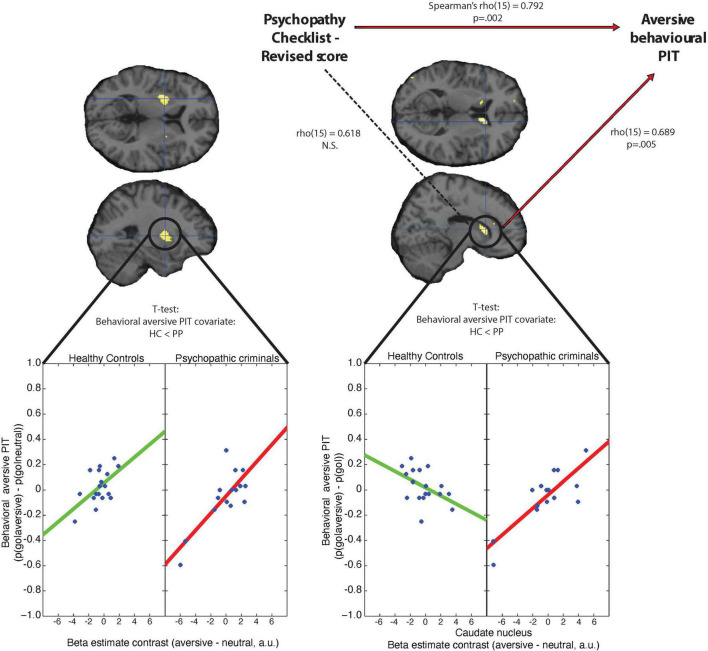
Association between aversive behavioral PIT, beta estimate contrasts (neutral–aversive trials) and psychopathy severity (in terms of the psychopathy checklist–revised total score) for the putamen and the caudate nucleus. **Left panel:** Beta estimate contrasts (neutral–aversive trials) within the putamen are positively correlated with behavioral aversive PIT [*p*(go| neutral)–*p*(go| aversive)] for violent offenders and healthy controls. **Right panel:** Beta estimate contrasts (neutral–aversive trials) within the caudate nucleus correlate positively with behavioral aversive PIT [*p*(go| neutral)–*p*(go| aversive)] for psychopathic criminals, but not for healthy controls. Correlations between the mean beta estimate contrasts and PCL-R and aversive behavioral PIT are calculated in terms of Spearman’s rho. Scatterplots are for illustrative purposes only and were created by plotting the behavioral PIT effect against the extracted average beta estimate contrast from the *p* < 0.001 whole-brain uncorrected clusters within the putamen and caudate nucleus.

Individual differences in behavioral aversive PIT [*p*(go| aversive)–*p*(go|neutral)] correlated positively with BOLD signal (aversive main regressor–neutral main regressor) in the putamen across both groups: Greater aversive inhibition of behavior was associated with reduced BOLD signal during aversive PIT trials (versus neutral) in the left putamen [Figure 3, peakvoxel MNI-coordinates (−26, 2, 6), *k* = 205, *Z* = 4.59, *p*_FWE_ = 0.004, small volume corrected]. In other words, higher BOLD signal in the putamen during the presentation of the aversive (versus neutral) Pavlovian cue was accompanied by greater disinhibition of go-actions, in line with the hypothesis that putamen signal reflects motor execution. Conversely, there was a significant difference between groups in the caudate nucleus [peakvoxel MNI-coordinates (14, 20, 10), *k* = 98, *Z* = 4.26, *p*_FWE_ = 0.006, small volume corrected, Figure 3]: By contrast to the putamen, signal in the caudate nucleus of healthy controls (aversive versus neutral) correlated negatively with aversive PIT [*p*_uncorrected_ = 0.012 at peak voxel (14, 18, 10), Figure 3], so that greater aversive inhibition of behavior was associated with greater aversive BOLD signal during aversive (versus neutral) PIT trials. This concurs generally with the hypothesis that caudate nucleus signal reflects the operation of a more complex computational operation related to motor planning (Alexander et al., 1986; Herz et al., 2013; Provost et al., 2015), such as the transfer of affective information onto action. Critically, this positive relation between BOLD signal in the caudate during aversive trials and behavioral inhibition was completely reversed in the violent offenders so that greater aversive inhibition of behavior in the violent offenders was associated with reduced BOLD signal during aversive (versus neutral) PIT trials in the caudate nucleus [peak voxel MNI-coordinates (14, 18, 10), *k* = 225, *Z* = 4.85, *p*_FWE_ = 0.039, corrected for the whole-brain]. Thus, the caudate nucleus of violent offenders acted like their putamen, perhaps reflecting a failure to engage the computation that is required for translating aversive information into action suppression. Note, that we repeated this correlation analysis within the violent offender group by means of Kendall’s tau analyses, because Kendall’s tau analysis is preferred over parametric methods for small samples and is robust to outliers (Croux and Dehon, 2010). Correlation of the mean beta estimates from the caudate nucleus with behavior remained significant [tau_(15)_ = 0.520, *p* = 0.008]. Moreover, based on reviewer comments we also conducted this analyses without the two violent offenders that visually might seem to drive these findings and found that the correlation is robust to excluding these data points [Tau_(13)_ = 0.450, *p* = 0.047].

**FIGURE 4:**
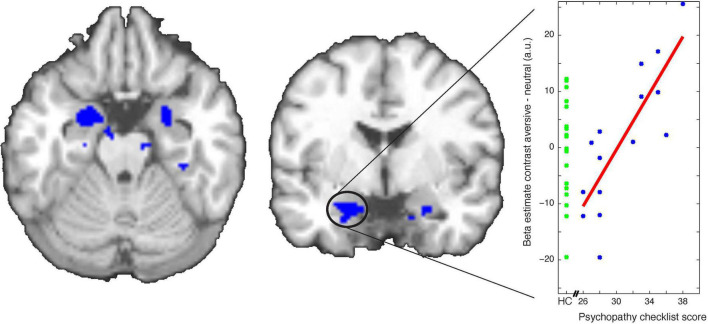
Association between psychopathy severity [in terms of Psychopathy Check List-Revised (PCL-R) total score] and beta estimate contrasts [conditioned stimuli (CS) aversive–CS neutral trials] during Pavlovian conditioning. Scatterplot depicts relation between PCL-R total score and the extracted average beta estimate contrast from the *p* < 0.001 whole-brain uncorrected cluster within the amygdala (for illustrative purpose only). Data is also displayed for healthy controls for whom no PCL-R score was available (green points).

Next, we asked whether the psychopathic tendency-related behavioral PIT effects (Figure 2B) were accompanied by psychopathic tendency-related differences in Pavlovian conditioning. Psychopathic tendency in terms of PCL-R score was indeed related to CS-dependent BOLD signal change (aversive versus neutral) over the whole conditioning stage) in the bilateral amygdala [right amygdala: peakvoxel MNI-coordinates (24 2 −24), *k* = 22, *Z* = 3.51, *p*_FWE_ = 0.033, left amygdala: peakvoxel MNI-coordinates (−18 −2 −22), *k* = 37, *Z* = 3.48, *p*_FWE_ = 0.036, small volume corrected for the bilateral amygdala; Figure 4]. This psychopathy severity dependent effect was also accompanied by group differences in the left amygdala [left amygdala: peakvoxel MNI-coordinates (−12 −2 −22), *k* = 5, *Z* = 3.65, *p* = 0.010, small volume corrected for the bilateral amygdala], so that violent offenders exhibited greater amygdala signal during aversive versus neutral cues compared with healthy controls. Note, that we did not find a significant association between CS-dependent amygdala signal during Pavlovian conditioning and aversive PIT behavior (*p*_uncorrected_ > 0.001).

## Discussion

The present study shows that, although we did not find behavioral group differences between healthy controls and violent offenders, higher psychopathic tendency within the violent offender sample was accompanied by attenuated inhibition of instrumental behavior in the presence of (non-consequential) Pavlovian aversive cues. In addition, while aversive PIT and caudate BOLD signal correlated negatively in healthy volunteers, this correlation was completely reversed in the violent offenders with psychopathy (compared with non-criminal healthy controls). Moreover, within the group of violent offenders, we established a positive association between psychopathic tendency and amygdala reactivity to aversive cues during Pavlovian conditioning. Together, these data suggest that psychopathic tendency is associated with enhanced aversive Pavlovian cue reactivity (cf. Schultz et al., 2016), yet reduced aversive inhibition of instrumental behavior. In addition, these data suggest a link between psychopathic tendency, Pavlovian aversive inhibition, and striatal action selection.

The finding that increased psychopathic tendency was associated with reduced aversive inhibition is reminiscent of findings from our previous work with a comparable experimental task, showing that central serotonin depletion reduced aversive inhibition in healthy volunteers (Geurts et al., 2013b). This observation is remarkable considering the prior finding that psychopathic tendency in a violent offender sample is accompanied by reduced serotonin metabolites in the cerebrospinal fluid (Soderstrom et al., 2001, 2003) and that callous-unemotional traits in boys was related to lower serum levels of serotonin (Moul et al., 2013). In line with these observations, we found a strong correlation between the PCL-R-score and aversive inhibition in a PIT task. The next step will be to assess whether aversive Pavlovian disinhibition in psychopathy can be countered by serotoninergic drugs, such as selective serotonin reuptake inhibitors, consistent with findings that provoked aggression in primary psychopathy can be reduced by serotonin augmentation by paroxetine (Fanning et al., 2014; e.g., Butler et al., 2021).

Our finding that the amygdala of violent offenders with high psychopathic tendency was more responsive to aversive (versus neutral) CSs than those with lower psychopathic tendency (Figure 4) is inconsistent with the hypothesis that violent offenders with high psychopathy severity scores would be insensitive to aversive cues (Flor et al., 2002; Birbaumer et al., 2005). This generally concurs with other prior findings that challenge the view that violent offenders with psychopathic tendency are insensitive to punishment and/or lack fear (e.g., Gregory et al., 2015; Hoppenbrouwers et al., 2016). More specifically our findings are in line with the largest study on Pavlovian conditioning in psychopathy that shows increased rather than reduced amygdala BOLD signal (Schultz et al., 2016) as well as recent meta-analyses of task-based activation studies in psychopathy that challenge the predominant view of amygdala hypo-reactivity in psychopathy (Deming and Koenigs, 2020). Remarkably, as was the case for central serotonin depletion (Geurts et al., 2013a) increased psychopathic tendency was associated with reduced aversive inhibition of both the approach and withdrawal actions. This suggests that the aversive transfer computation that is disrupted operates at the level of action intensity rather than of action valence.

Both healthy volunteers and violent offenders exhibited a negative between-participant correlation between the degree to which instrumental actions were inhibited by aversive cues and the degree to which BOLD signal in the putamen was activated during aversive cues. This observation is generally in line with putamen signal reflecting motor execution (Alexander et al., 1986; Herz et al., 2013; Provost et al., 2015). By contrast, signal in the caudate nucleus of healthy volunteers correlated positively with the degree to which instrumental actions were inhibited by aversive cues. Thus, greater aversive inhibition was associated with greater caudate nucleus signal during aversive cues. This raises the possibility that signal in the caudate nucleus of healthy controls does not reflect motor execution *per se*, but rather reflects the operation of the aversive Pavlovian inhibition computation of interest. The key finding is that this association was completely reversed in the violent offenders, so that the across-participant pattern of caudate nucleus signal resembled that in their putamen. In line with evidence from work with experimental rodents, evidence in humans (Balleine and O’Doherty, 2009) implicates the caudate nucleus [together with the ventromedial prefrontal cortex (Valentin et al., 2007)] in the instrumental control of behavior (Tanaka et al., 2008; de Wit et al., 2012) as well as response inhibition (Watanabe and Munoz, 2010; Schmidt et al., 2020). Accordingly, one might speculate, based on our neural findings, that aversive Pavlovian disinhibition in psychopathy is accompanied by a failure of the caudate nucleus to exhibit the Pavlovian inhibition computations that it exhibits normally, as suggested by the negative relation in healthy controls. Instead, the caudate nucleus of violent offenders exhibited the across-participant pattern of effects seen in their putamen, which instead reflects increases in motor execution: greater signal with more Go actions. We note that the group differences in the continuous brain-behavior associations were not accompanied by group differences in average BOLD signal. This suggests that the behavioral deficits in violent offenders do not reflect a failure to recruit the caudate nucleus *per se*, but rather that they reflect a failure to recruit the caudate nucleus as a function of the relevant aversive inhibition computation.

One puzzle is that the range of behavioral PIT scores in the violent offenders was comparable with that in the controls. Thus, we did not provide a conceptual replication of the expected group effect on behavior found in our previous study (Ly et al., 2016): While we did find a relation between psychopathic tendency and attenuated aversive inhibition within the group of violent offenders, we did not observe the impact of aversive cues on instrumental action to be altered in the group of violent offenders compared with the group of non-violent healthy controls. This complicates the interpretation of our results. One implication is that abnormal aversive PIT *per se* is not a sufficient prerequisite for developing criminal psychopathic tendency. Criminal psychopathy might surface only if abnormal aversive PIT is accompanied, for example, by excessive impact of reward on behavior and cognition (Buckholtz et al., 2010; Bjork et al., 2012; Yildirim and Derksen, 2015; Geurts et al., 2016). This would be in line with the literature on increased reward seeking and decreased sensitivity to punishment in psychopathic individuals (e.g., Newman et al., 1990). Thus, it might be that one factor or an interaction between multiple factors moderates the impact of differences in aversive PIT on clinical symptomatology (cf. Plichta and Scheres, 2014 for explanation of the moderator model). PCL-R-scores from the healthy controls were not available and therefore we cannot exclude that a similar association exists in healthy controls. However, we think this is unlikely, because there were no correlations between scores on the Psychopathic Personality Inventory and aversive PIT (Supplementary results).

We consider several explanations for this lack of a group effect. First, we have a relatively small sample size to detect such a group difference, which might have led to false negative findings. Second, our paradigm is not optimized for indexing appetitive PIT. As the main finding of Ly et al. (2016) is based on a contrast between aversive and appetitive cues, this effect might be due to aberrant effects of violent offenders to either appetitive, aversive or both cues. In the current study, focusing on aversive versus neutral cues, we did not find this group difference. This raises the possibility that the findings of Ly et al. (2016) reflect combined modulation of appetitive biasing as well as aversive biasing. Unfortunately, the insensitivity of our paradigm to appetitive PIT (cf. Geurts et al., 2013a; see Supplementary results) precludes strong conclusion about the valence-specificity of the effects. Third, where Ly et al. (2016) use happy and sad faces as affective cues that are presented during instrumental learning, we use neutral stimuli associated with appetitive and aversive juices that we present during already learned instrumental trials presented in nominal extinction. It might be that these latter Pavlovian CS sort more effect in violent offenders than (sad) faces (cf. Brennan and Baskin-Sommers, 2021). Future studies comparing effects of facial expressions with Pavlovian CS on instrumental behavior might elucidate these discrepancies.

Finally, we highlight the following limitations of the current study: First, although our sample size is comparable to that of other neuroimaging studies focusing on psychopathic criminals (cf. Brazil et al., 2009; von Borries et al., 2009; Rothemund et al., 2012; Contreras-Rodríguez et al., 2014), we recognize the limitation of such a small sample size especially when considering our parametric analysis (Button et al., 2013). We did find statistically significant, robust effects, but future studies with larger sample sizes are necessary to confirm the relationships uncovered in this study. Second, we were not able to replicate the strong action-specific BOLD signal found in the ventromedial prefrontal cortex as observed in our previous fMRI study with the same paradigm in healthy young volunteers (Geurts et al., 2013a). We have recently replicated this ventromedial effect in young women (both healthy and with borderline personality disorder) in another independent dataset (submitted to this issue, D. E. M. Geurts, T. J. van den Heuvel, R. Cools). One factor that might account for this discrepancy is that the average performance at the end of the instrumental task was significantly better in the latter studies (with mainly graduate students) compared with that of the healthy controls in the current study [mean accuracy (SEM): 2013 study = 0.76 (0.023), current = 0.64 (0.027), *t*-test: *t*_36_ = 3.6, *p* = 0.001]. Thus, it is possible that the healthy controls and patients in the current study relied to a lesser degree on a goal-directed control strategy and to a greater degree on a habitual control strategy than did the subjects in our previous study. Although speculative, this might explain why the putamen was recruited as a function of aversive PIT in the current study in both the healthy control group and the psychopathy group, which was not the case in our previous study. Relevant in this context might also be the fact that the current study included only men, whereas the other studies mainly included women. Third, we should note that our group comparison is necessarily confounded by overt criminal history. As such, we cannot and do not claim specificity of our findings to violent offenders with psychopathic tendency compared with non-psychopathic violent offenders or psychopathic non-offenders (cf. “successful psychopaths”).

In sum, our results strengthen the hypothesis that psychopathic tendency in violent offenders is associated with abnormal impact of aversive Pavlovian suppression of instrumental behavior. The neural results raise the possibility that this reflects deficits in neural computations involving the caudate nucleus.

## Data availability statement

The raw data supporting the conclusions of this article will be made available by the authors, without undue reservation.

## Ethics statement

The studies involving human participants were reviewed and approved by Medisch Ethische Toetsing Commissie (METC) Oost-Nederland. The patients/participants provided their written informed consent to participate in this study.

## Author contributions

DG and KB collected the data. DG performed the statistical analyses under supervision of RC. DG and RC wrote the first draft of the manuscript. All authors contributed to conception and design of the study and manuscript revision, read, and approved the submitted version.
