# Supplementary material for: Psychopathic tendency in violent offenders is associated with reduced aversive Pavlovian inhibition of behavior and associated striatal BOLD signal
Source: Front Behav Neurosci. 2022 Oct 14;16:963776. doi: 10.3389/fnbeh.2022.963776 (PMC9614330; doi:10.3389/fnbeh.2022.963776)
Supplement: Supplementary file 1 [file Data_Sheet_1.docx]

Supplementary Material

- 1. **Supplementary Material and Methods**
  2. **Forensic psychiatric hospital**

The Pompestichting is a "TBS-clinic" located in Nijmegen. TBS (“Ter Beschikking Stelling”) is a treatment disposal on behalf of the state for people who committed serious criminal offences in connection with having a mental disorder. TBS is not a punishment, but an entrustment act for mentally disordered offenders (diminished responsibility). These court orders are an alternative to either long term imprisonment or confinement in psychiatric hospital, with the goal to strike a balance between security, treatment and protection.

- 1. **Additional procedural details**

Participants received written and oral information about the experiment and signed an informed consent. All participants were invited for a screening session and a scan session with no more than two weeks in between the appointments. During the first appointment, they were screened for psychiatric exclusion criteria by trained psychologists using the Structure Clinical Interview for DSM disorders to exclude axis 2 disorder (SCID-II; Dutch version, Weertman (Weertman et al., 2000)), Mini International Neuropsychiatric Interview to exclude axis 1 disorder (MINI; Dutch version(van Vliet et al., n.d.)) and the Dutch version of the National Adult Reading Test for IQ assessment (NLV, Schmand (1991)). Psychiatric exclusion criteria were recent major depressive disorder, bipolar disorder, schizophrenia, schizoaffective disorder, schizophreniform disorder, delusional and other psychotic disorders, schizoid or schizotypical personality disorder, current alcohol and substance intoxication, first degree relatives with DSM IV axis I schizophrenia or schizophreniform disorder.

Further, participants completed the Psychopathy Personality Inventory (PPI) (Lilienfeld and Andrews, 1996; Dutch version: Jelicic et al., 2004). They were instructed not to drink more than 3 units/day during in the week preceding the experimental measure; not to use of alcohol within 24 hours of the measurement; not to use cannabis or other illicit drugs within the week before measurement; not to use psychotropic medication other than oxazepam during the 5 days before measurement; not to use oxazepam within 12 hours before measurement; and not to smoke within 1 hour before measurement and no more than five cigarettes on the scan day. Furthermore, they were asked to refrain from any caffeinated drinks and chocolate on the scan day and to refrain from extensive physical exercise and heavy meals before the scan session. In the scanner, participants wore earplugs with integrated speakers. Foam pads were placed inside the head coil and paper tape was placed over the forehead and the base of the head coil to restrict movement. Before performing the PIT task, participants performed an approach avoidance task and monetary incentive delay task reported elsewhere (Borries et al., 2012; Geurts et al., 2016). After a break of 15 minutes, they were seated in front of a laptop and they clicked through the same instructions they would receive within the scanner. The investigator who sat next to the participants during these instructions answered possible questions. Instructions and task images were then projected onto a translucent screen at the end of the scan tube, which was visible via a mirror attached to the head coil. After again displaying the instructions of the task, the task was started, which lasted about 50 minutes.

- 1. **Additional task details**

The paradigm was programmed using Matlab® (2009b, TheMathWorks, Natick, MA) with the Psychophysical Toolbox extension(Brainard, 1997).

- - 1. **Additional information on the instrumental stage**

To orthogonalize the approach-withdrawal and appetitive-aversive axes, the learned instrumental values in approach and withdrawal needed to be matched. To achieve this, both go and no-go responses were, if correct, rewarded to the same extent. Additionally, to avoid confound of behavioural activation, in each condition (i.e., in both approach and withdrawal conditions) the go response was designated as the correct response for half of the instrumental stimuli, and the no-go response for the other half. Incorrect responses had opposite outcome contingencies to correct responses, yielding more punishments than rewards. This ensured that go, no-go, approach, and withdrawal overall had the same learned association with rewards and punishments. In both the approach and withdrawal action context, there were two go stimuli, which yielded reward more often after active responses (and punishment after not responding), and two no-go stimuli, which yielded reward more often after not responding (and punishment after go responding) (on average the ratio reward:punishment after a correct action was 0.86:0.14 for go-stimuli and 0.84:0.16 for nogo-stimuli). Trials were labelled as correct if subjects chose the usually rewarded response. Average reinforcement was matched between approach and withdrawal contexts (mean proportion of positively reinforced trials for approach = 0.59; for withdrawal =0.57, paired sample T-test: T_33_=1.3, p=.2). Accordingly, the instrumental stimuli in the approach and withdrawal conditions did not differ in acquired value, so that any differences between conditions cannot reflect differential Pavlovian responses elicited by these instrumental stimuli. Rather than representing effects of competing Pavlovian responses, the effects we report represent PIT effects, i.e., the effects of Pavlovian CSs on instrumental behaviour in terms of choice (percentage of go-choices) and vigour (average number of button presses on go-trials). Initial stimuli and action context were randomized across participants.

- 1. **Additional information on image acquisition**

Whole-brain imaging was performed on a 3-Tesla MR scanner (Magnetrom Trio Tim, Siemens Medical Systems, Erlangen, Germany). Functional data were obtained using a multiecho gradient T2*-weighted EPI (ME-EPI) scanning sequence with BOLD contrast (38 axial-oblique slices; repetition time = 2.32 sec; echo times = 9.0, 19.3, 30, and 40 msec; in plane resolution = 3.3 × 3.3 mm; slice thick- ness = 2.5 mm; distance factor = 0.17; flip angle = 90°, 194 volumes per run acquired during the PIT stage). Visual stimuli were projected on a screen and were viewed through a mirror attached to the head coil. In addition, a high-resolution T1-weighted magnetization- prepared rapid-acquisition gradient-echo anatomical scan was obtained from each participant (192 sagittal slices; repetition time = 2.3 sec; echo time = 3.03 msec; voxel size = 1.0 × 1.0 × 1.0 mm; field of view = 256 mm).

1. **Supplementary Results**
   1. **Instrumental stage**

Subjects learned to make correct choices during the instrumental learning stage indicated by an increasing number of correct responses across time (main effect of Time: F_1;31_=27.4.0, p<.001) (Supplementary figure 1A). There was a significant Action Context x Response Type interaction (F_1;31_=60.6, p <.001). This was driven by an effect in the approach Action Context where subjects performed better on approach-go stimuli compared with approach-nogo stimuli (F_1;31_=36.7, p<.001), whereas in the withdrawal Action Context subjects performed better on withdrawal-nogo stimuli compared with withdrawal-go stimuli (F_1;31_=6.6, p=.015). In addition, across Time subjects made more correct go responses than correct nogo responses (main effect of Response Type: F_1;31_=4.5, p=.045).

Performance at the end of instrumental training generalized to and persisted throughout the PIT stage (Supplementary figure 1): there were no significant main effects of, or interactions with a Time factor with 3 levels: the end of the instrumental training, the beginning of the PIT stage and the end of the PIT stage (F_2;62_<1.5, p>.2). Other patterns found during the instrumental training remained significant during the PIT stage (main effect of Response Type: F_1;31_=12.6, p=.001; Action Context x Response Type interaction: F_1;31_=13.7, P =.001). There were no significant interactions with or a main effect of the factor Group (F_2,62_<2.5, p>.12). Critically, there were also no performance differences between groups when the PIT stage was analysed by itself (main effect of Group: F_1;31_=2.0, P =.17).

- - 1. **No relation between performance in the instrumental stage and aversive PIT**

In order to analyse whether performance (i.e., accuracy, p(correct)) during the instrumental stage influenced the outcome of the PIT stage, (1) the performance at the end of the instrumental training and (2) the difference in performance between the beginning and the end of the instrumental training were added as covariates to the rmANOVA (Group x Action Context x Valence). If anything, adding these covariates increased the significance of the action-specific aversive PIT effect across both groups and neither resulted in any interactions with performance.

- 1. **Pavlovian stage**

Three violent offenders were excluded from the analysis of the query trials, because they failed to answer more than half of the query trials in time (2 sec). Results from the analysis of our primary (behavioural and imaging) effects of interest did not change when we excluded the subjects who did not complete the query trials. Furthermore, there was no significant correlation between PCL-R score on the one hand and performance on the query trials or number of missed query trials on the other hand (Spearman’s rho_13_ = .54, p=.19). There was also no performance difference between groups on the Pavlovian query trials, suggesting that explicit CS-US associations were unaffected (Mann-Whitney U test: mean proportion correct across blocks in violent offenders (n=13): 85%; SEM: 4.9; range: 50-100%; HCs(n=18): 88%; SEM: 3.4; range: 63-100%, p=1.0).

Data for the VAS-rating analysis were not available for one healthy control subject (had to leave earlier) and 3 violent offenders (one who only finished the first block and two due to technical error at the second post-conditioning rating). VAS (liking) ratings for the Pavlovian CSs showed that the Pavlovian conditioning procedure induced changes in subjective liking and that there were no differences between the groups: The aversive CS became more aversive (Wilcoxon Signed Rank Test: p<.001) and the neutral CS did not change (Wilcoxon Signed Rank Test: p=.22). After conditioning the aversive CS was judged to be more aversive than the neutral (Mann-Whitney U test: p=.001). None of the VAS ratings for the Pavlovian CSs or their changes from before to after conditioning differed between the groups (All Mann-Whithney U tests: p>.19). These VAS ratings suggest robust Pavlovian conditioning that does not significantly differ between groups. None of the (changes in) VAS ratings were significantly related to the PCL-R score.

Mann-Whitney U tests showed that there were no differences between the groups in how they rated the aversive juice before and after conditioning (for all comparisons p>.61).

- - 1. **Results were robust to exclusion of subjects with aberrant performance on query trials**

After excluding three subjects who failed to respond to most of the query trials the correlation between PCL-R and aversive Pavlovian inhibition remained strong and significant: Spearman rank correlation: rho_12_ = -.730, p=.007. This held also for the correlation between PCL- R and mean betas from the caudate nucleus: Spearman rank correlation: rho_12_ = -.67, p=.017; and for the correlation between PCL-R and mean betas form the putamen for the whole group: rho_30_=.49, p=.006.

Further significant contrasts and their reported correlations from the imaging analysis did not change substantially when excluding these subjects.

- 1. **Pavlovian to instrumental transfer stage**
     1. **Vigour**

Analysis of vigour (i.e. number of button presses) revealed a main effect of Valence (neutral vs aversive) (Supplementary table 3, F_1;31_=10.8, p=.002). There were no significant differences between the groups in terms of the vigour of responding and the effect of CS Valence on vigour did not depend on individual differences in clinical PCL-R ratings (Valence x PCL-R: F_(1,13)_=3.0, p=.106).

- - 1. **Psychopathy Personality Inventory does not interact with PIT**

In addition to PCL-R scores (obtained only in violent offenders) we obtained PPI scores in both groups. We note that we did not find any significant association between behavioural aversive PIT and either the PPI total score or the 2-factor model subscores (all p>.1) (across both groups, and in each group separately).

- - 1. **Insensitivity to appetitive PIT**

In supplementary analyses, we confirmed that the paradigm was not sensitive to appetitive PIT (neutral vs appetitive). The rmANOVA with Action Context (approach/withdrawal) and CS Valence (neutral/appetitive) as within subject factors and Group (HCs/violent offenders) as between subject factor did not reveal any significant PIT-effects not with choice (p(go), all F<1.7, all p>.05) and not with vigour (number of button presses, all F<3.4, all p>.05) as dependent variable.

# Supplementary Figures and Tables

## Supplementary Figures

**Supplementary figure 1** Instrumental learning and generalization to the Pavlovian-instrumental transfer stage for healthy controls (left panel) and violent offenders (right panel). The proportion of correct choices (p(correct)) are broken down by response type (go/nogo) and action context (approach/withdrawal). Error bars represent standard errors of the mean.

- 1. **Supplementary Tables**

Supplementary table 1 Visual analogue scale ratings before and after Pavlovian conditioning (1=very nice, 0=very aversive, mean [SEM]).

|  | Healthy controls (n=20) | | Violent offenders (n=15) | |
| --- | --- | --- | --- | --- |
|  | Before | After | Before | After |
| Appetitive | .52 (.040) | .60 (.026) | .52 (.048) | .61 (.032) |
| Neutral | .47 (.046) | .52 (.033) | .49 (.056) | .49 (.040) |
| Aversive | .53 (.039) | .34 (.044) | .48 (.047) | .38 (.053) |

**Supplementary table 2** *Choice (p(go) as a function of ACTION context and CS VALENCE (mean [SEM]).*

|  | Healthy controls (n=20) | | Violent offenders (n=16) | |
| --- | --- | --- | --- | --- |
|  | Approach | Withdrawal | Approach | Withdrawal |
| Appetitive | .652 (.037) | .498 (.042) | .773 (.047) | .551 (.047) |
| Neutral | .647 (.037) | .525 (.048) | .748 (.042) | .588 (.053) |
| Aversive | .578 (.043) | .623 (.044) | .676 (.048) | .609 (.050) |

**Supplementary table 3** *Number of button presses during go-trials as a function of ACTION context and CS VALENCE (mean [SEM]).*

|  | Healthy controls (n=20) | | Violent offenders (n=16) | |
| --- | --- | --- | --- | --- |
|  | Approach | Withdrawal | Approach | Withdrawal |
| Appetitive | 7.73 (.44) | 7.43 (.53) | 8.74 (.49) | 7.86 (.59) |
| Neutral | 7.82 (.40) | 7.88 (.51) | 8.49 (.45) | 8.00 (.57) |
| Aversive | 7.23 (.51) | 7.78 (.50) | 8.09 (.57) | 8.00 (.56) |
